# Supplementary material for: A Humanized Yeast Phenomic Model of Deoxycytidine Kinase to Predict Genetic Buffering of Nucleoside Analog Cytotoxicity
Source: Genes (Basel). 2019 Sep 30;10(10):770. doi: 10.3390/genes10100770 (PMC6826991; doi:10.3390/genes10100770)

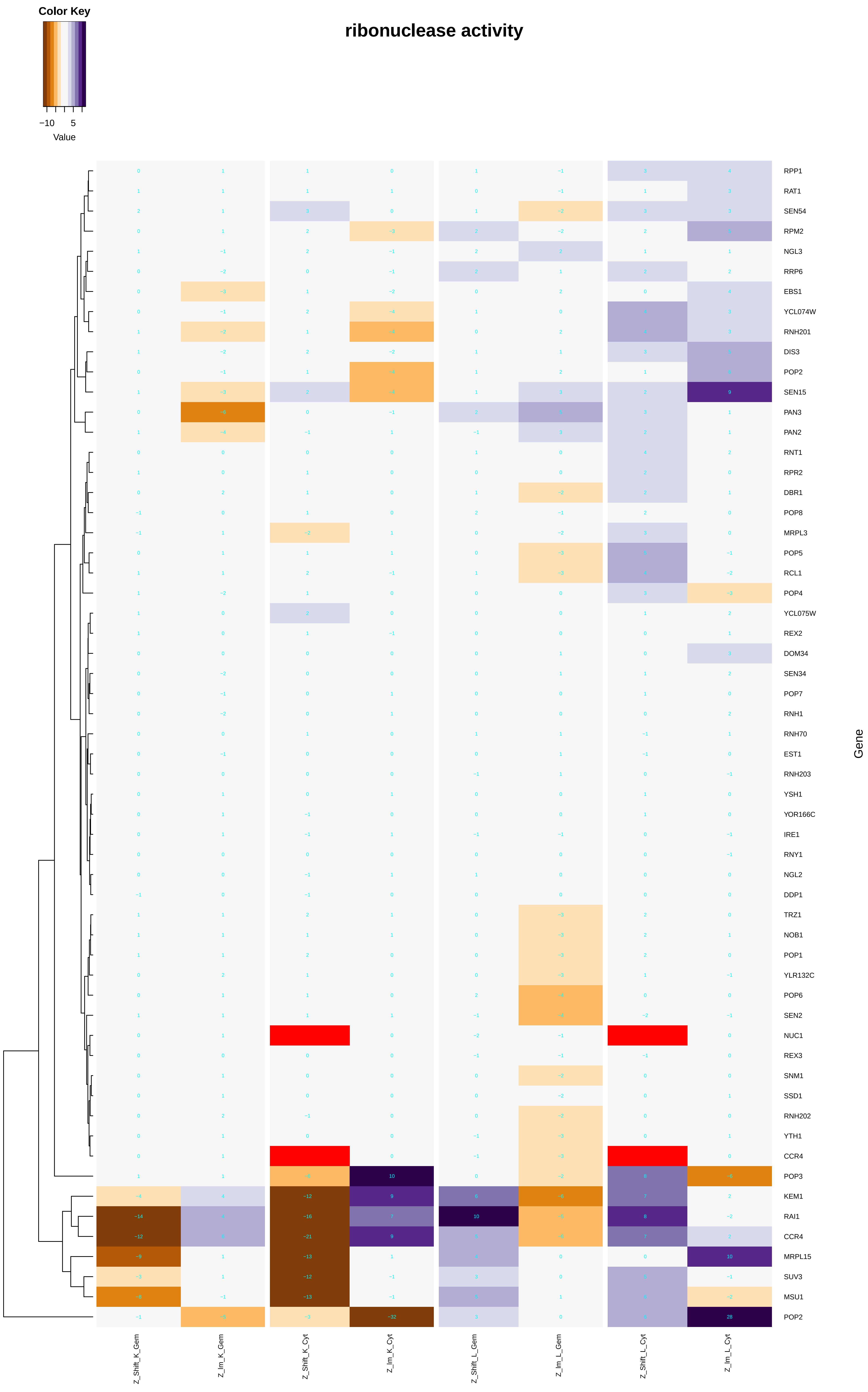
ribonuclease activity
Color Key
−10
5
Value
Gene
RPP1
RAT1
SEN54
RPM2
NGL3
RRP6
EBS1
YCL074W
RNH201
DIS3
POP2
SEN15
PAN3
PAN2
RNT1
RPR2
DBR1
POP8
MRPL3
POP5
RCL1
POP4
YCL075W
REX2
DOM34
SEN34
POP7
RNH1
RNH70
EST1
RNH203
YSH1
YOR166C
IRE1
RNY1
NGL2
DDP1
TRZ1
NOB1
POP1
YLR132C
POP6
SEN2
NUC1
REX3
SNM1
SSD1
RNH202
YTH1
CCR4
POP3
KEM1
RAI1
CCR4
MRPL15
SUV3
MSU1
POP2
Z_Shift_K_Gem
Z_Im_K_Gem
Z_Shift_K_Cyt
Z_Im_K_Cyt
Z_Shift_L_Gem
Z_Im_L_Gem
Z_Shift_L_Cyt
Z_Im_L_Cyt

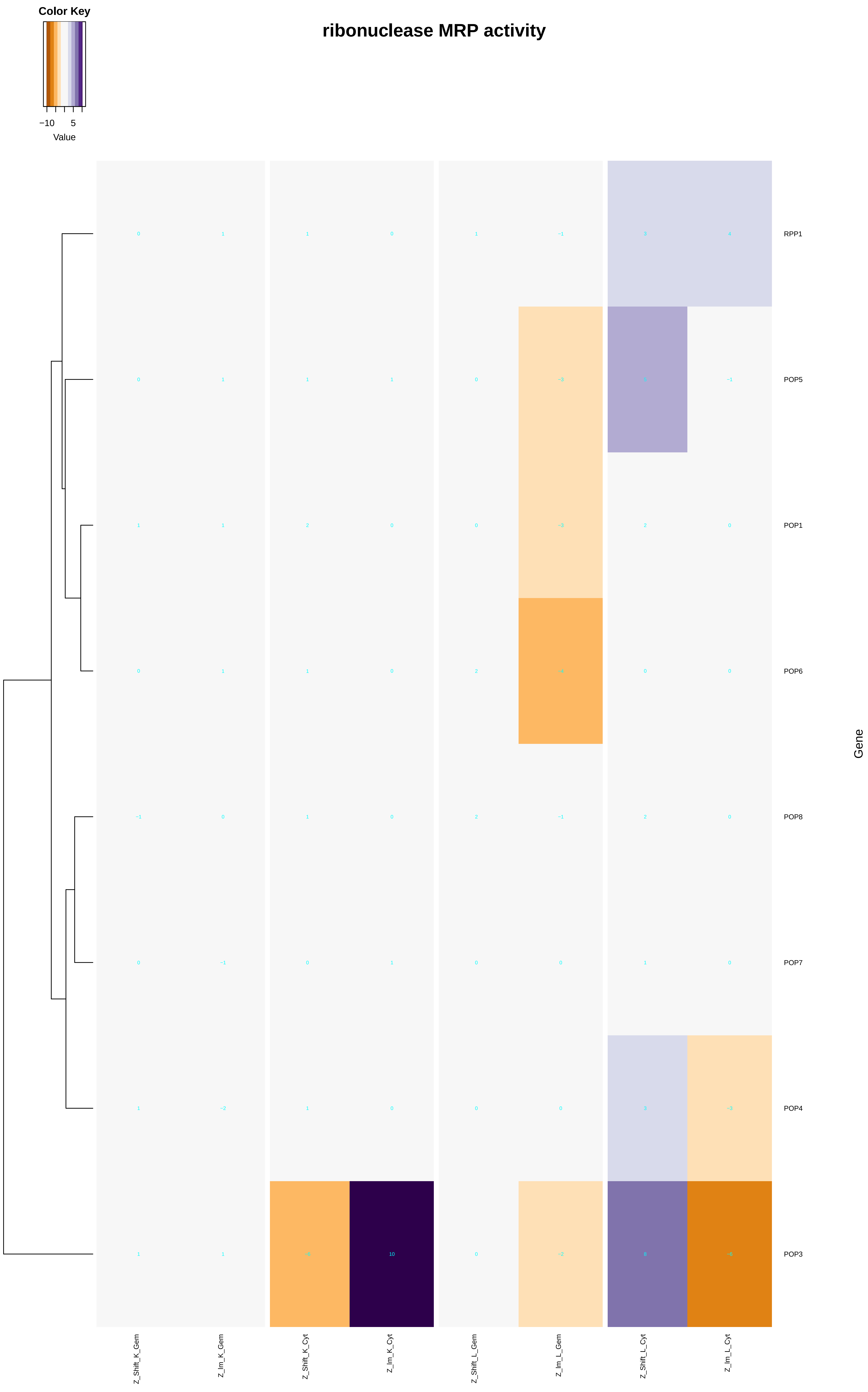

ribonuclease MRP activity
Color Key
−10
5
Value
RPP1
POP5
POP1
POP6
POP8
POP7
POP4
POP3
Gene
Z_Shift_K_Gem
Z_Im_K_Gem
Z_Shift_K_Cyt
Z_Im_K_Cyt
Z_Shift_L_Gem
Z_Im_L_Gem
Z_Shift_L_Cyt
Z_Im_L_Cyt

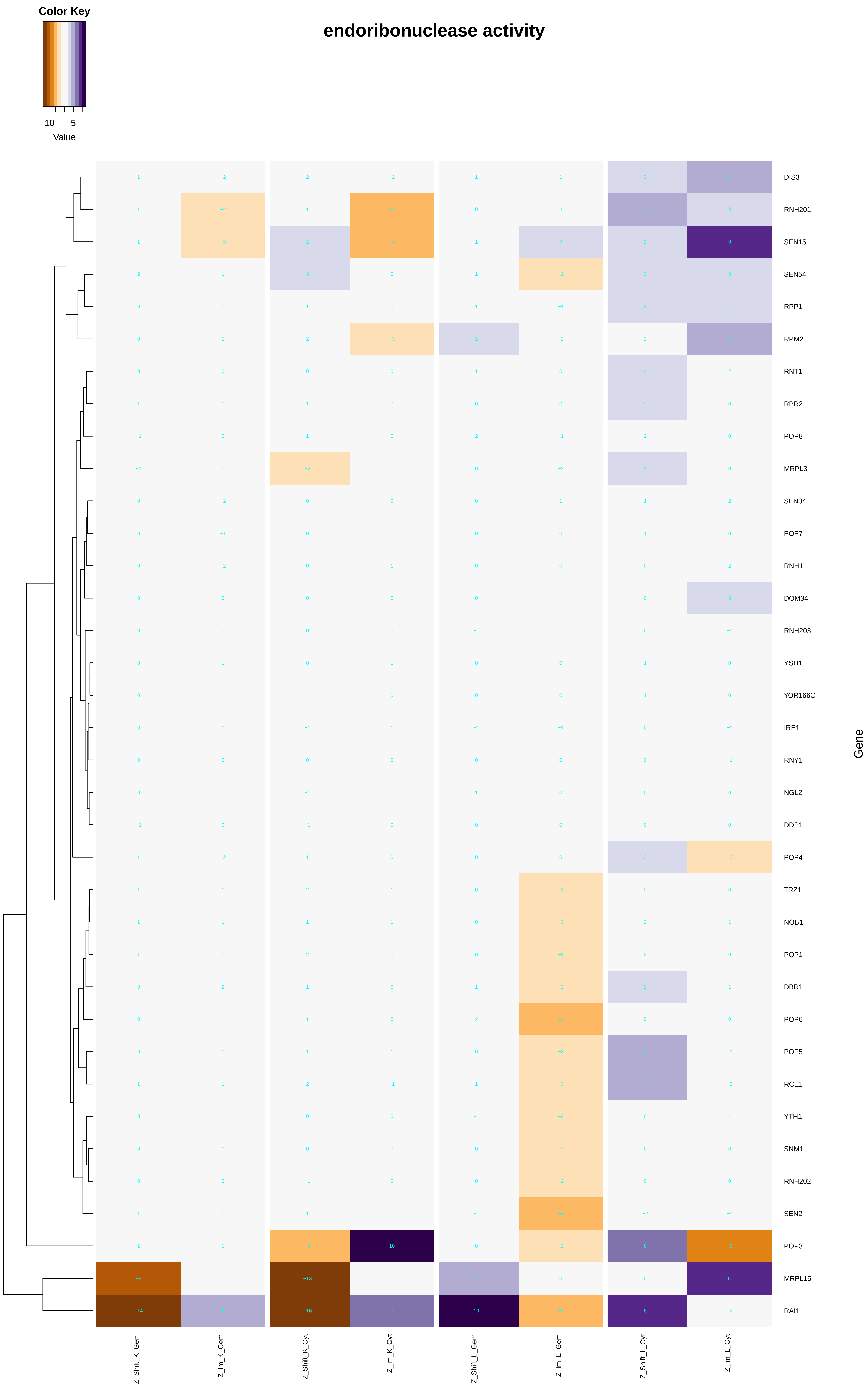

Color Key
−10
5
Value
endoribonuclease activity
DIS3
RNH201
SEN15
SEN54
RPP1
RPM2
RNT1
RPR2
POP8
MRPL3
SEN34
POP7
RNH1
DOM34
RNH203
YSH1
YOR166C
IRE1
RNY1
NGL2
DDP1
POP4
TRZ1
NOB1
POP1
DBR1
POP6
POP5
RCL1
YTH1
SNM1
RNH202
SEN2
POP3
MRPL15
RAI1
Gene
Z_Shift_K_Gem
Z_Im_K_Gem
Z_Shift_K_Cyt
Z_Im_K_Cyt
Z_Shift_L_Gem
Z_Im_L_Gem
Z_Shift_L_Cyt
Z_Im_L_Cyt

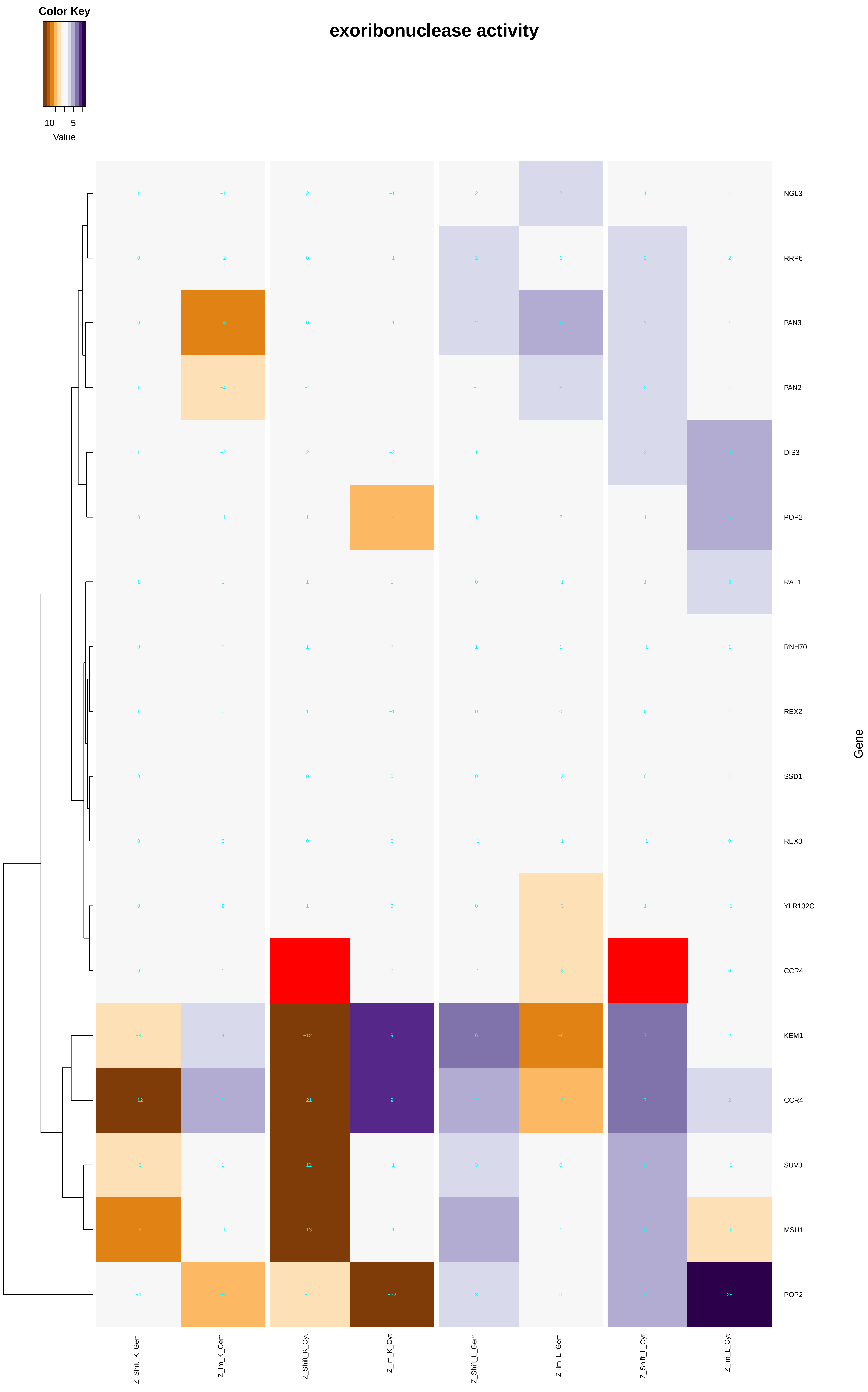

Color Key
−10
5
Value
exoribonuclease activity
NGL3
RRP6
PAN3
PAN2
DIS3
POP2
RAT1
RNH70
REX2
SSD1
REX3
YLR132C
CCR4
KEM1
CCR4
SUV3
MSU1
POP2
Gene
Z_Shift_K_Gem
Z_Im_K_Gem
Z_Shift_K_Cyt
Z_Im_K_Cyt
Z_Shift_L_Gem
Z_Im_L_Gem
Z_Shift_L_Cyt
Z_Im_L_Cyt

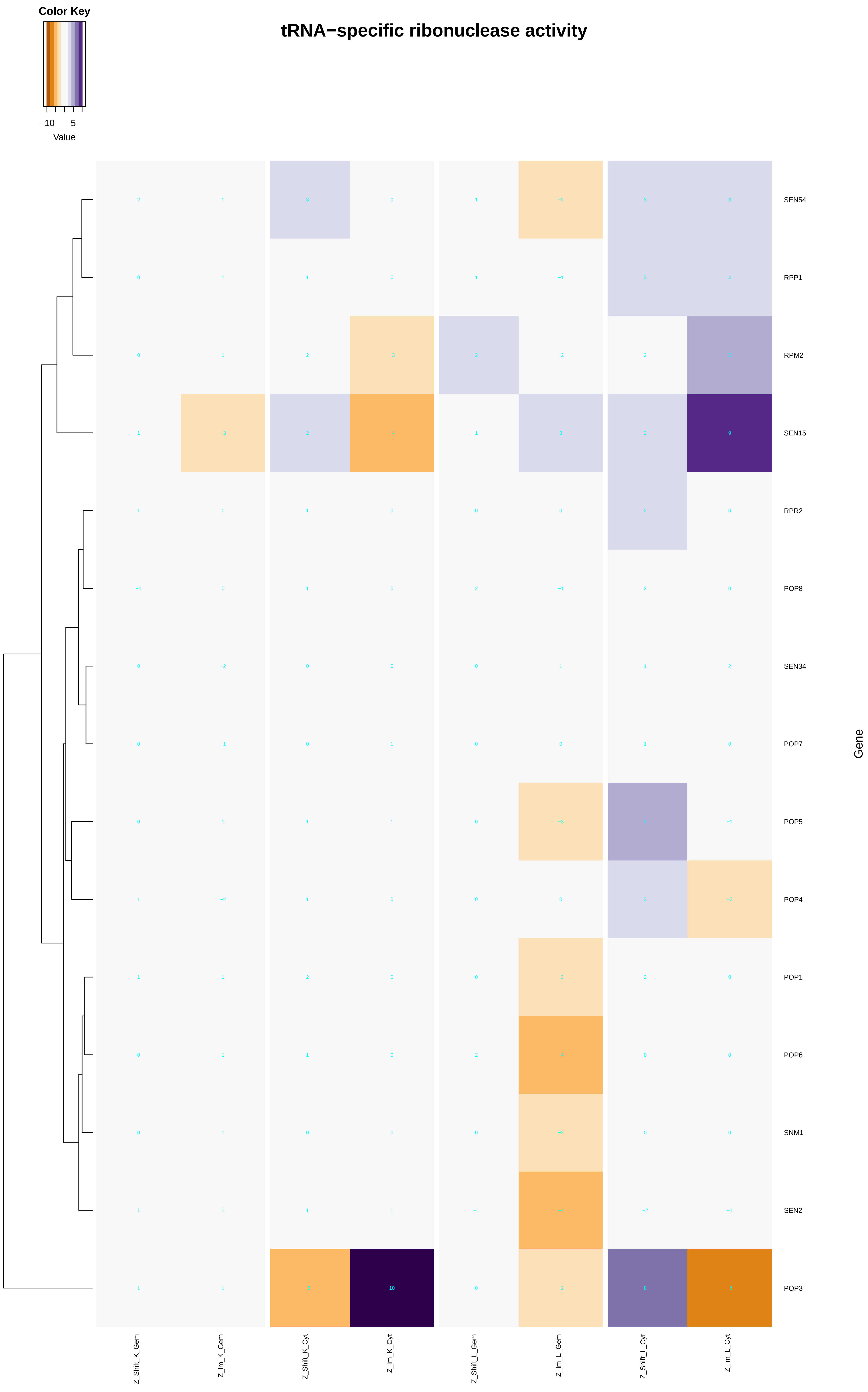

Color Key
−10
5
Value
tRNA−specific ribonuclease activity
SEN54
RPP1
RPM2
SEN15
RPR2
POP8
SEN34
POP7
POP5
POP4
POP1
POP6
SNM1
SEN2
POP3
Gene
Z_Shift_K_Gem
Z_Im_K_Gem
Z_Shift_K_Cyt
Z_Im_K_Cyt
Z_Shift_L_Gem
Z_Im_L_Gem
Z_Shift_L_Cyt
Z_Im_L_Cyt

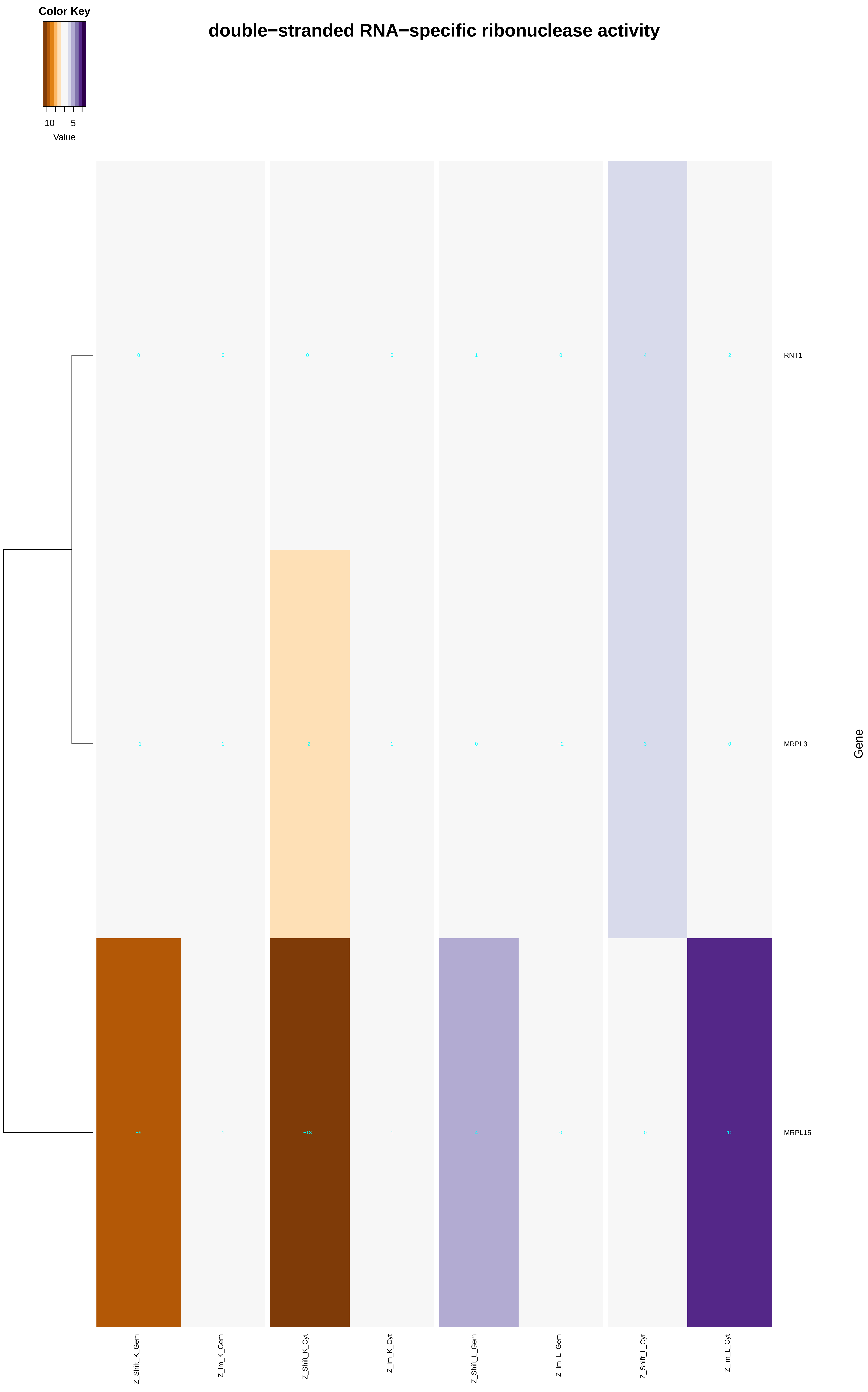
Color Key
−10
5
Value
double−stranded RNA−specific ribonuclease activity
RNT1
MRPL3
MRPL15
Gene
Z_Shift_K_Gem
Z_Im_K_Gem
Z_Shift_K_Cyt
Z_Im_K_Cyt
Z_Shift_L_Gem
Z_Im_L_Gem
Z_Shift_L_Cyt
Z_Im_L_Cyt

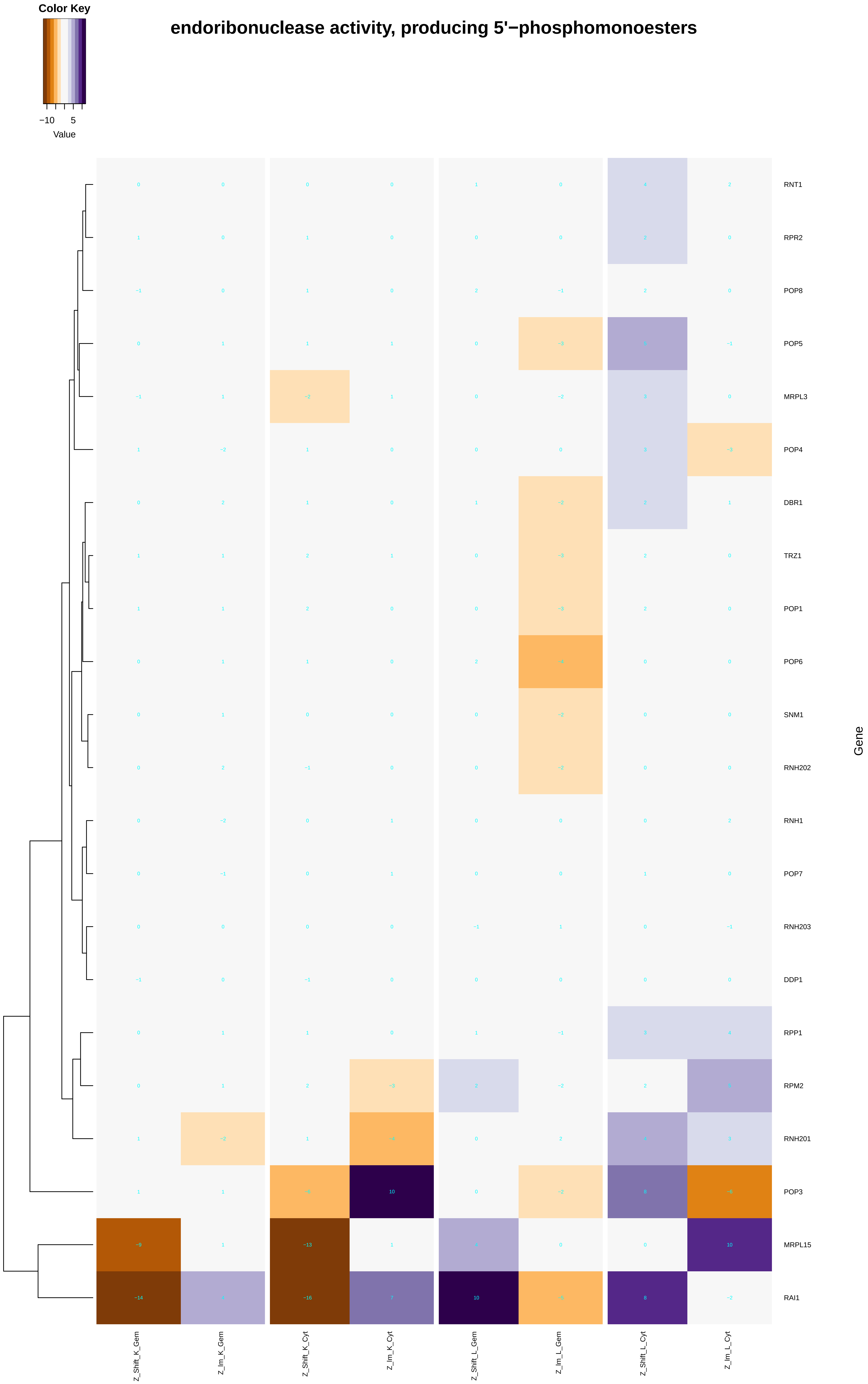

endoribonuclease activity, producing 5'−phosphomonoesters
Color Key
−10
5
Value
RNT1
RPR2
POP8
POP5
MRPL3
POP4
DBR1
TRZ1
POP1
POP6
SNM1
RNH202
RNH1
POP7
RNH203
DDP1
RPP1
RPM2
RNH201
POP3
MRPL15
RAI1
Gene
Z_Shift_K_Gem
Z_Im_K_Gem
Z_Shift_K_Cyt
Z_Im_K_Cyt
Z_Shift_L_Gem
Z_Im_L_Gem
Z_Shift_L_Cyt
Z_Im_L_Cyt

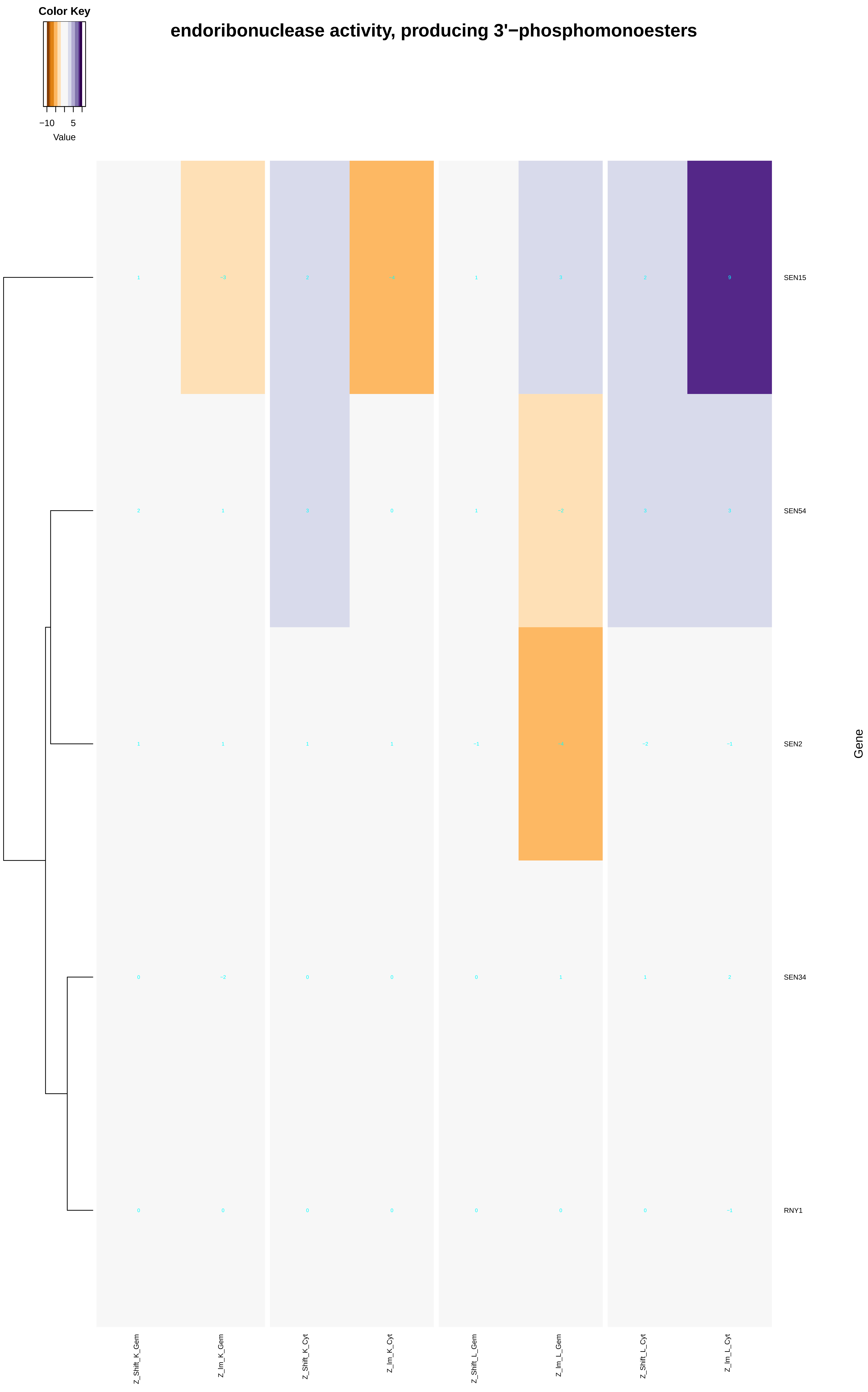
endoribonuclease activity, producing 3'−phosphomonoesters
Color Key
−10
5
Value
SEN15
SEN54
SEN2
SEN34
RNY1
Gene
Z_Shift_K_Gem
Z_Im_K_Gem
Z_Shift_K_Cyt
Z_Im_K_Cyt
Z_Shift_L_Gem
Z_Im_L_Gem
Z_Shift_L_Cyt
Z_Im_L_Cyt

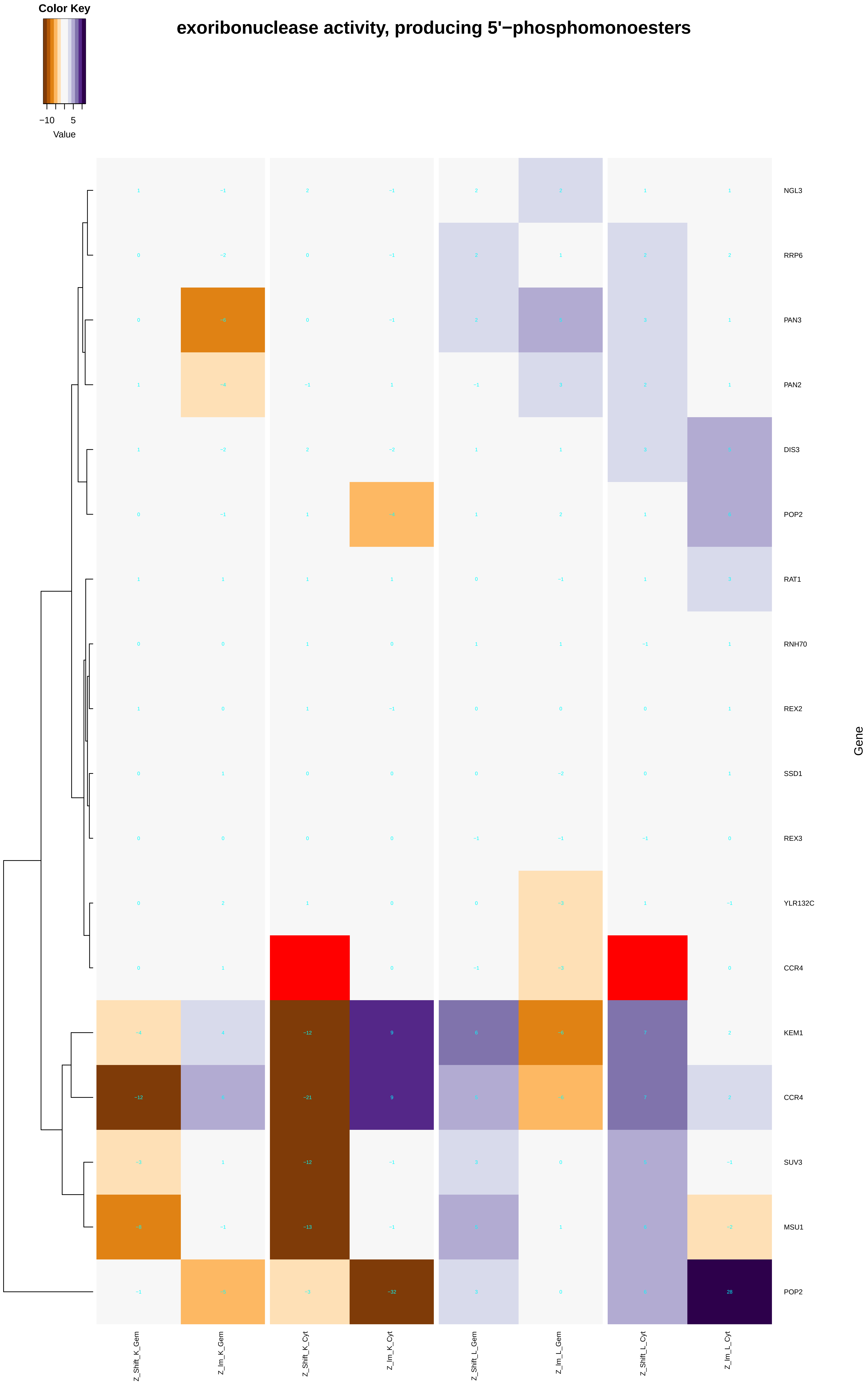

Color Key
−10
5
Value
exoribonuclease activity, producing 5'−phosphomonoesters
NGL3
RRP6
PAN3
PAN2
DIS3
POP2
RAT1
RNH70
REX2
SSD1
REX3
YLR132C
CCR4
KEM1
CCR4
SUV3
MSU1
POP2
Gene
Z_Shift_K_Gem
Z_Im_K_Gem
Z_Shift_K_Cyt
Z_Im_K_Cyt
Z_Shift_L_Gem
Z_Im_L_Gem
Z_Shift_L_Cyt
Z_Im_L_Cyt

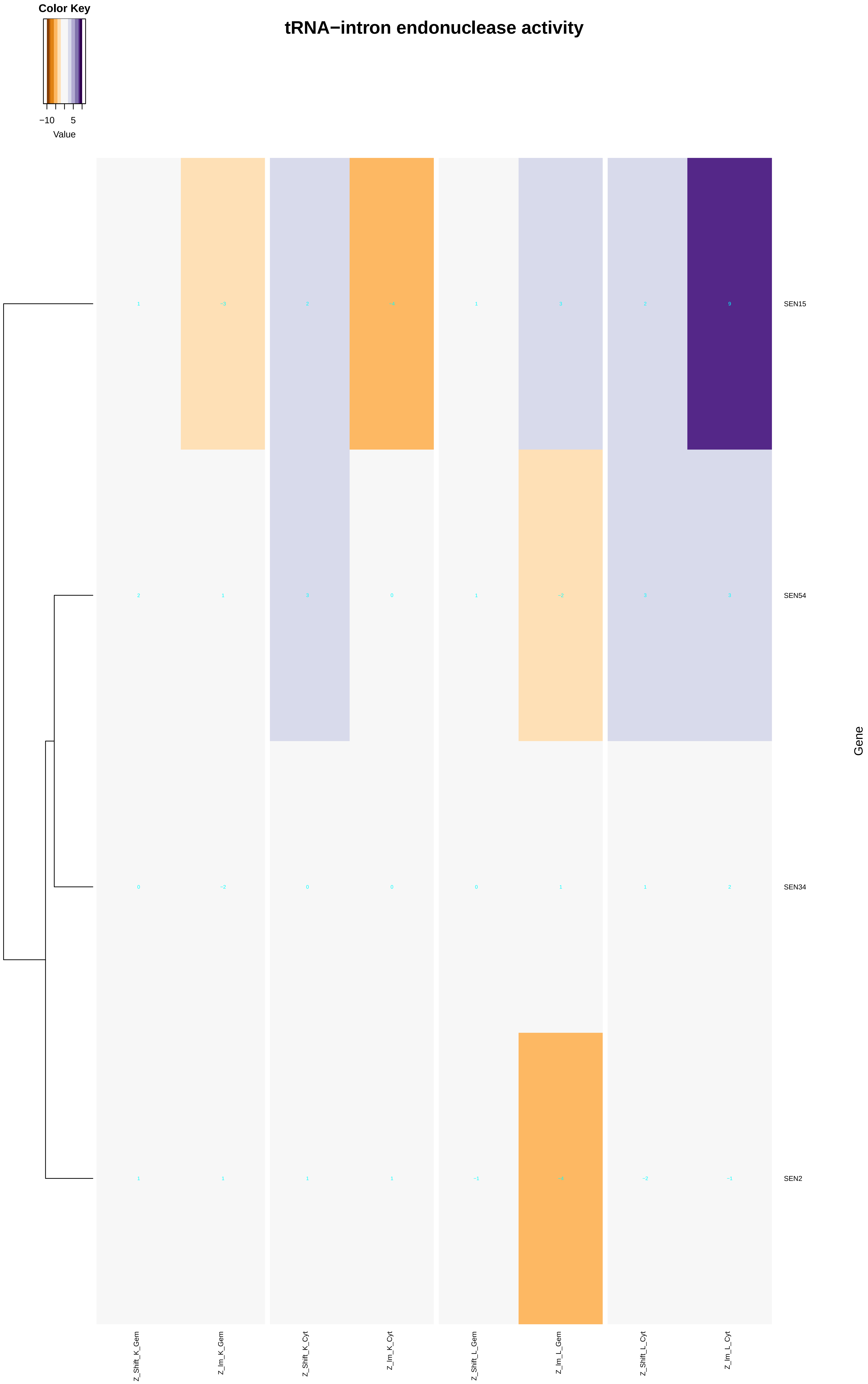
Color Key
−10
5
Value
tRNA−intron endonuclease activity
1
−3
2
−4
1
3
2
9
SEN15
2
1
3
0
1
−2
3
3
SEN54
0
−2
0
0
0
1
1
2
SEN34
1
1
1
1
−1
−4
−2
−1
SEN2
Gene
Z_Shift_K_Gem
Z_Im_K_Gem
Z_Shift_K_Cyt
Z_Im_K_Cyt
Z_Shift_L_Gem
Z_Im_L_Gem
Z_Shift_L_Cyt
Z_Im_L_Cyt

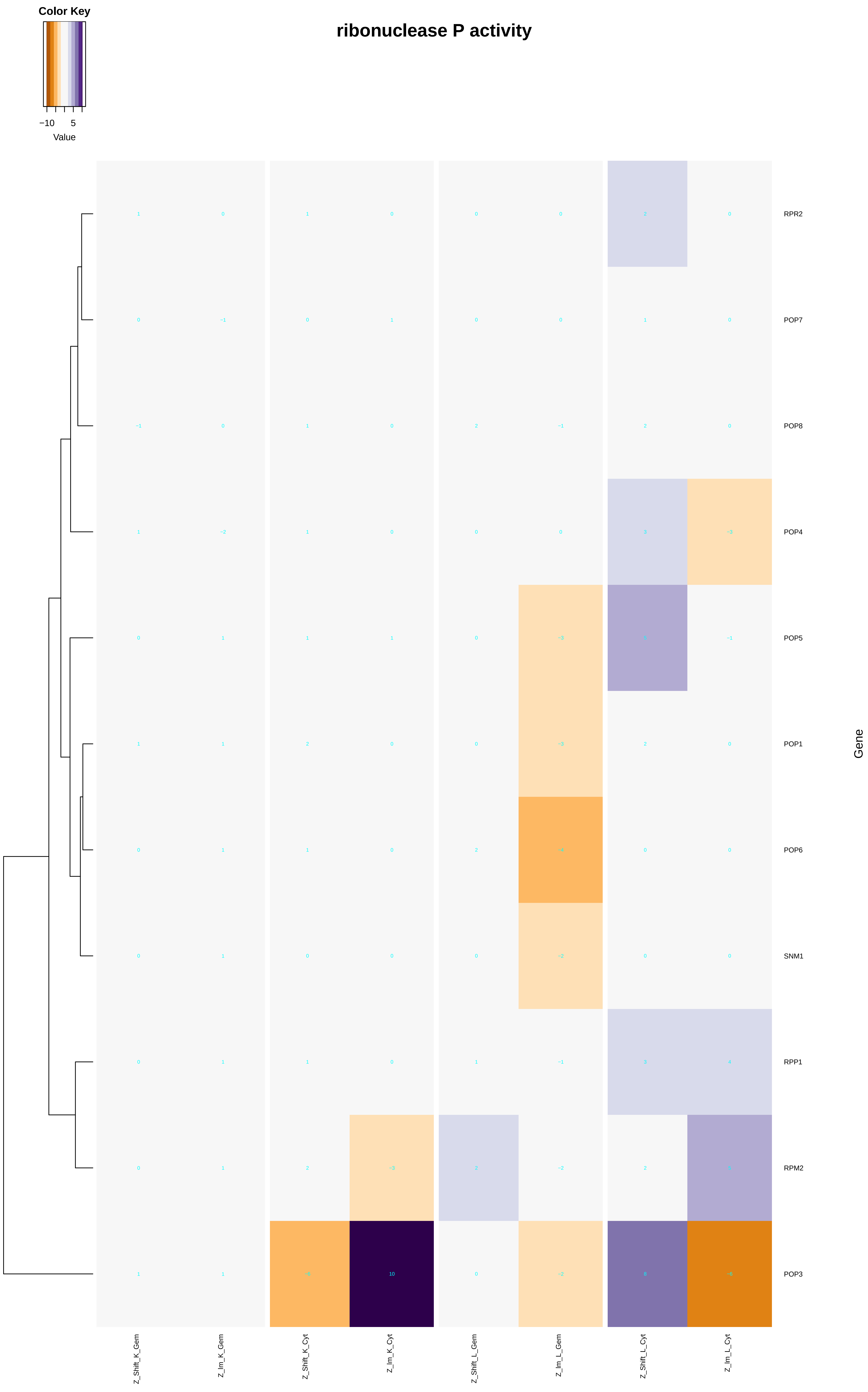

ribonuclease P activity
Color Key
−10
5
Value
RPR2
POP7
POP8
POP4
POP5
POP1
POP6
SNM1
RPP1
RPM2
POP3
Gene
Z_Shift_K_Gem
Z_Im_K_Gem
Z_Shift_K_Cyt
Z_Im_K_Cyt
Z_Shift_L_Gem
Z_Im_L_Gem
Z_Shift_L_Cyt
Z_Im_L_Cyt

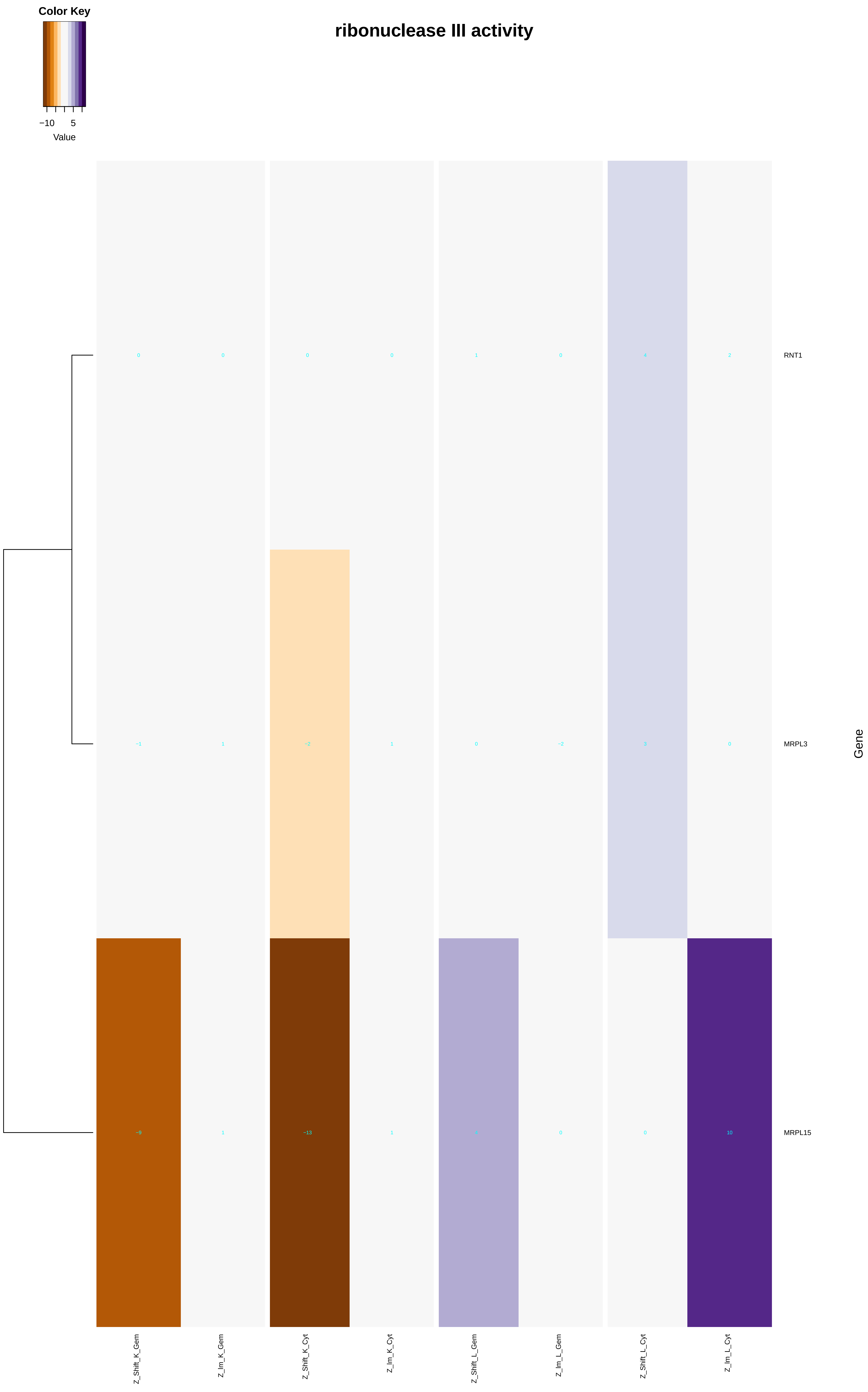
ribonuclease III activity
Color Key
−10
5
Value
RNT1
MRPL3
MRPL15
Gene
Z_Shift_K_Gem
Z_Im_K_Gem
Z_Shift_K_Cyt
Z_Im_K_Cyt
Z_Shift_L_Gem
Z_Im_L_Gem
Z_Shift_L_Cyt
Z_Im_L_Cyt

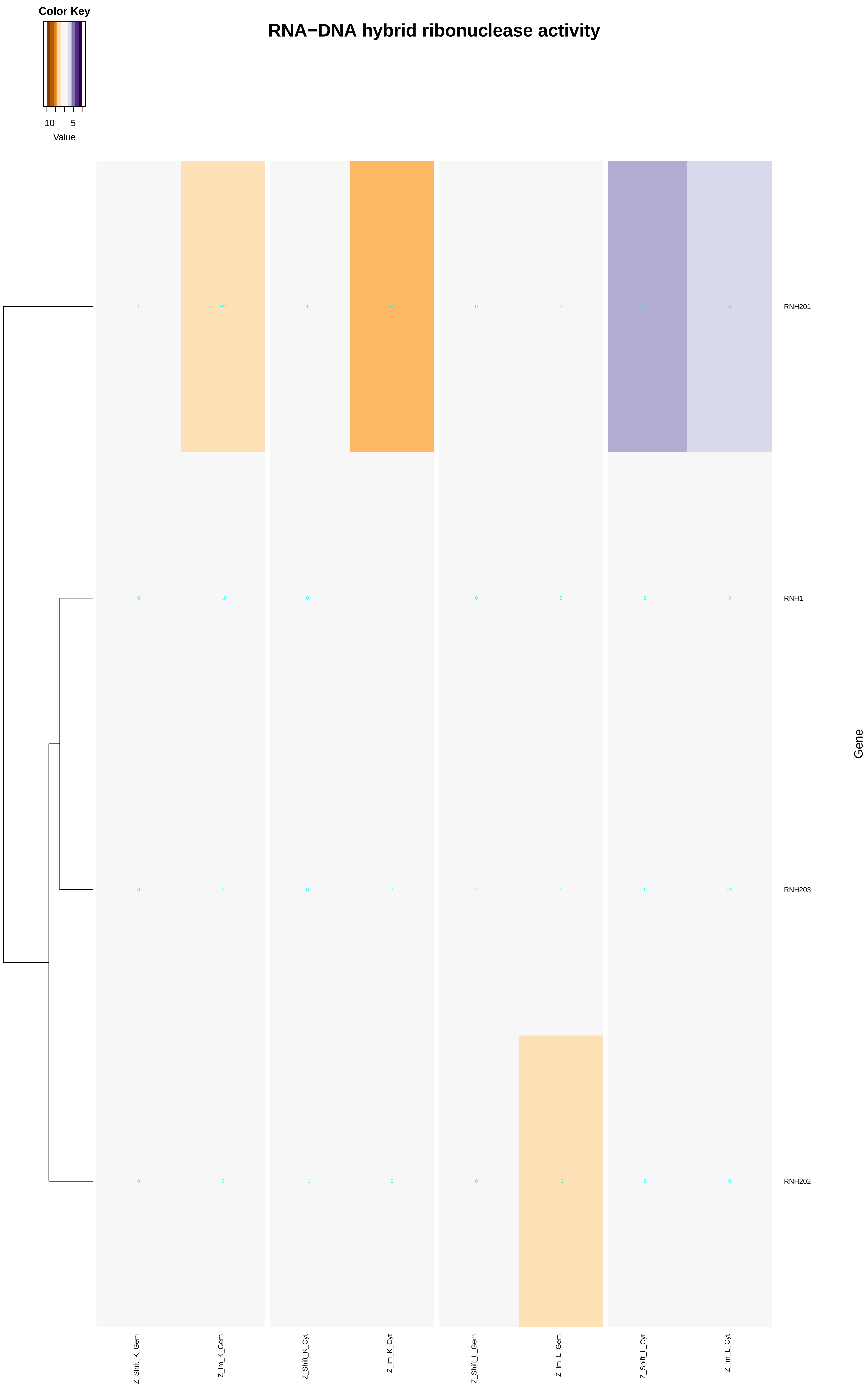
Color Key
−10
5
Value
RNA−DNA hybrid ribonuclease activity
RNH201
RNH1
RNH203
RNH202
Gene
Z_Shift_K_Gem
Z_Im_K_Gem
Z_Shift_K_Cyt
Z_Im_K_Cyt
Z_Shift_L_Gem
Z_Im_L_Gem
Z_Shift_L_Cyt
Z_Im_L_Cyt

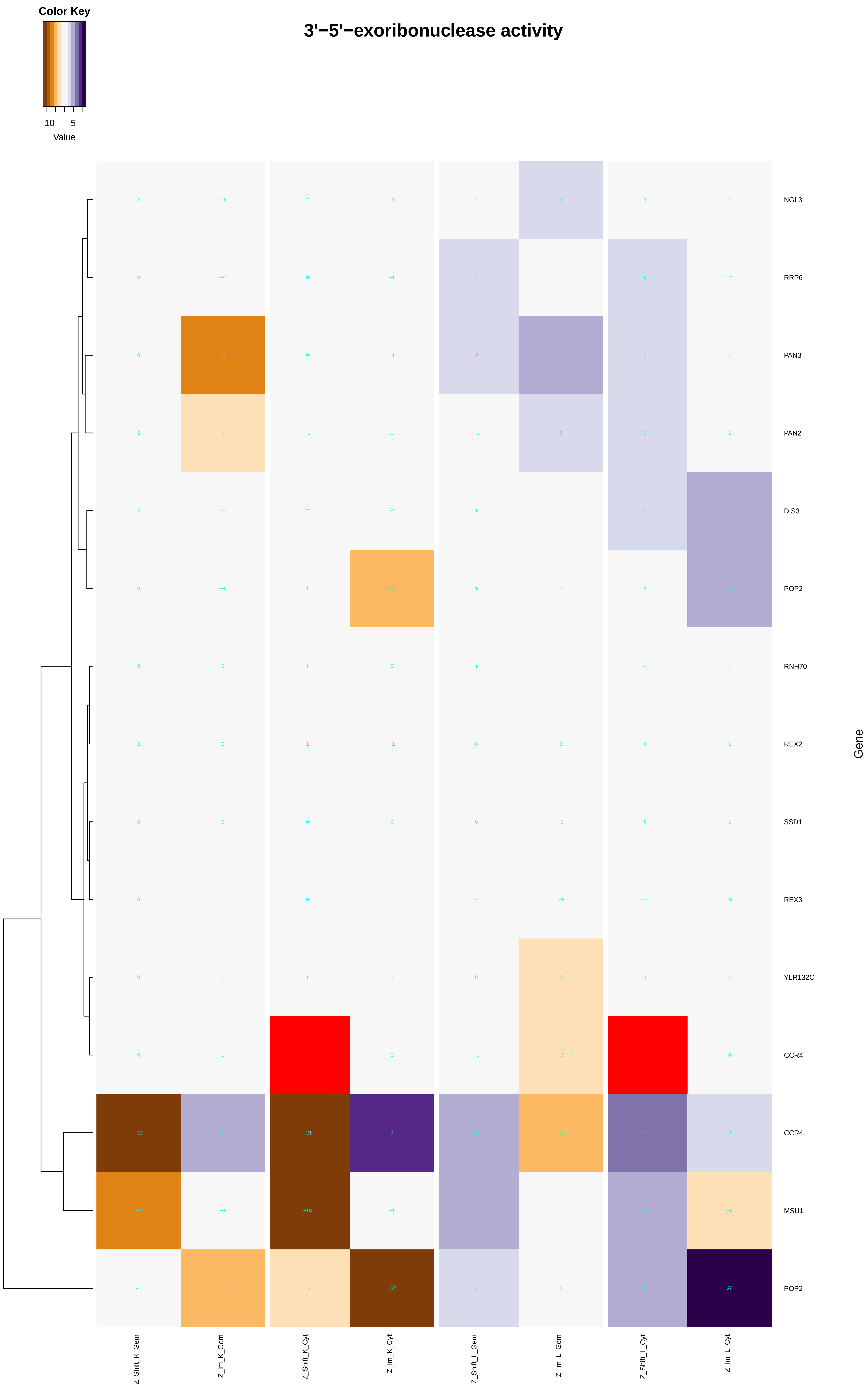

3'−5'−exoribonuclease activity
Color Key
−10
5
Value
NGL3
RRP6
PAN3
PAN2
DIS3
POP2
RNH70
REX2
SSD1
REX3
YLR132C
CCR4
CCR4
MSU1
POP2
Gene
Z_Shift_K_Gem
Z_Im_K_Gem
Z_Shift_K_Cyt
Z_Im_K_Cyt
Z_Shift_L_Gem
Z_Im_L_Gem
Z_Shift_L_Cyt
Z_Im_L_Cyt

poly(A)−specific ribonuclease activity

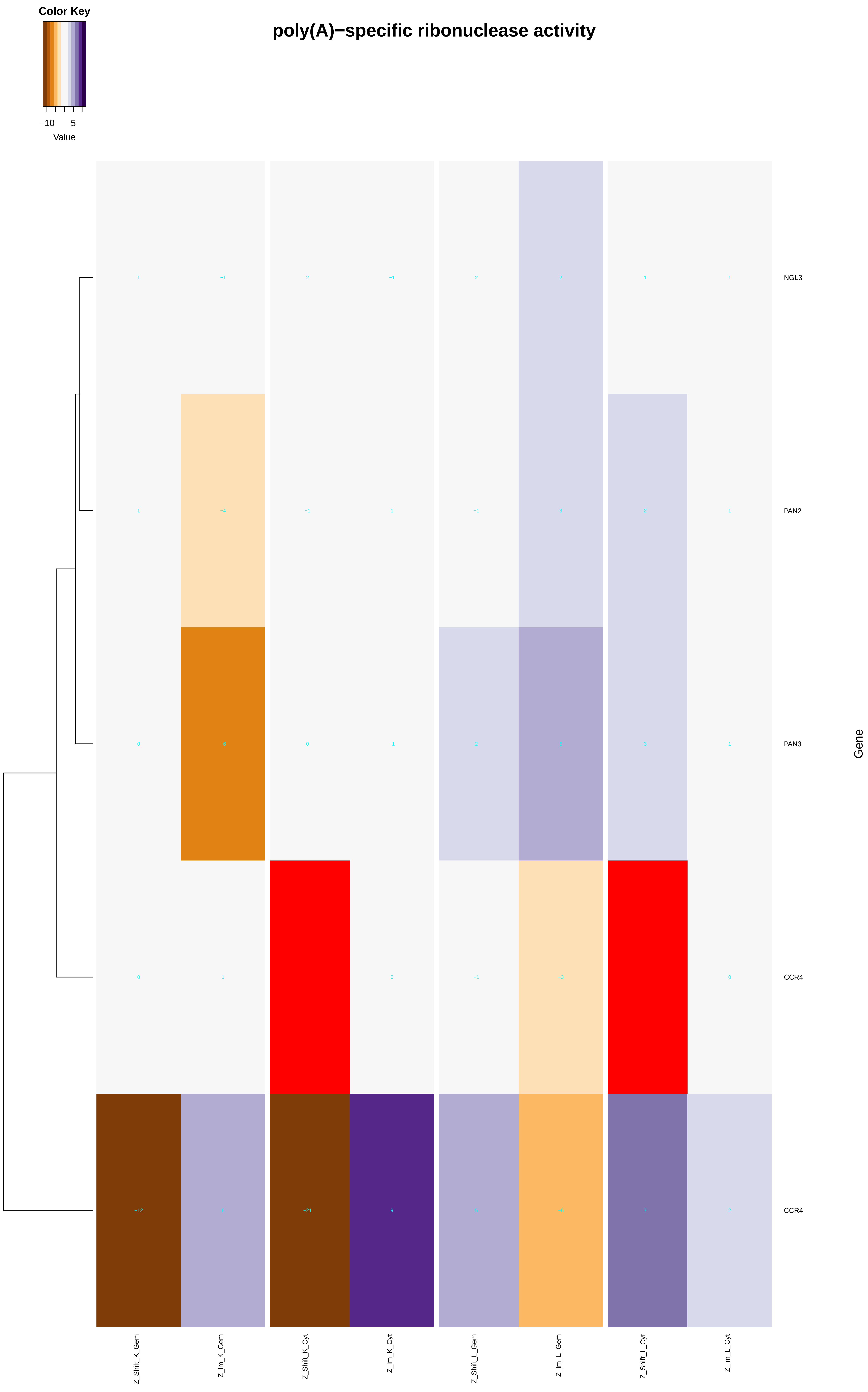

Supplement: Supplementary file 1 [file genes-10-00770-s001.zip › Additional_Files/Additional_File7_GOTermHeatmaps.zip!/Additional_File7_GOTermHeatmaps/ribonuclease_activity.pdf]
